# Clinical Features and Long Term Outcome of 102 Treated Autoimmune Hepatitis Patients

**DOI:** 10.5812/hepatmon.808

**Published:** 2012-02-29

**Authors:** Zinab Malekzadeh, Sepideh Haghazali, Sadaf G. Sepanlou, Homayoon Vahedi, Shahin Merat, Masoud Sotoudeh, Siavosh Nasseri-Moghaddam, Reza Malekzadeh

**Affiliations:** 1 Digestive Disease Research Center, Shariati Hospital, Tehran University of Medical Sciences, Tehran, IR Iran; 2 Sasan Alborz Biomedical Research Institute, Tehran, IR Iran; 3 Department of Pathology, Shariati Hospital, Tehran University of Medical sciences, Tehran, IR Iran

**Keywords:** Hepatitis, Autoimmune, Natural History, Liver Cirrhosis, Hepatitis, Chronic, Iran

## Abstract

**Background:**

There is limited data on the natural history of autoimmune hepatitis (AIH) and on the long-term follow-up of AIH patients who have been referred for regular medical attention.

**Objectives:**

We evaluated the clinical presentation and natural history of AIH in a large cohort of type I AIH patients from Iran.

**Patients and Methods:**

Between 1997 and 2008, 102 patients were enrolled in the study. Patients were diagnosed using the International Autoimmune Hepatitis Group criteria and were followed up for an average of 60 months. Clinical and biochemical data were gathered from all the patients at both the beginning and the end of the follow-up period. Liver biopsy was performed in all patients before treatment, and the biopsies were performed in 28 patients after treatment.

**Results:**

Biochemical remission was achieved by 80 (79.4%) patients. Of these, 53 (66.5%) showed near-normal liver histology or liver function test results and sonogram. The remaining 27 (33.5%) patients also achieved clinical and biochemical remission, but developed compensated cirrhosis. After a period of remission, 24 patients (32.5%) relapsed. Among the 22 (21.6%) patients who showed ultimate treatment failure, 6 underwent orthotopic liver transplantation and 3 died of liver failure while awaiting a transplant. Sixteen (72.7%) of the 22 patients who did not respond to therapy were non-compliant with medications and had irregular follow-up. The overall 10-year survival rate in the cohort was 96%.

**Conclusions:**

Long-term survival in AIH patients is very good. Prompt diagnosis and appropriate first-line and salvage therapy that includes close follow-up will make liver transplantation a rare necessity in the treatment of this disease.

## 1. Background

Autoimmune hepatitis (AIH) is a progressive disease of unknown cause that is characterized by chronic hepatitis, abnormal liver enzyme levels, high serum gamma globulin levels, and circulating autoantibodies [1][2]. It is more common in women than in men (sex ratio, 3.6:1) and is found in all age and ethnic groups [1][2]. There are no epidemiological data on the incidence and prevalence of AIH in Iran and other Middle Eastern countries, but an annual incidence of 2 per 100,000 and a point prevalence of 15 per 10,000 persons have been reported in the Caucasian populations of Northern European countries [1][2]. The uncontrolled immune response in AIH patients is associated with a decrease in the number and function of T-regulatory cells, resulting in immune intolerance and emergence of liver-targeted autoimmunity. This leads to a progressive necroinflammatory and fibrotic process in the liver [2][3]. Two major forms of AIH, type 1 and type 2, have been described on the basis of their autoantibody profiles [4]. Overlap syndromes with primary sclerosing cholangitis (PSC) or primary biliary cirrhosis (PBC) also occur [4]. The diagnosis of AIH is based on clinical, histopathological, and paraclinical findings. The International Autoimmune Hepatitis Group (IAIHG) has recently suggested revised criteria for use in research and simplified criteria for clinical use [5][6][7]. The AIH mortality rate is 80% in untreated patients [1]. Prednisolone, alone or in combination with azathioprine, is the standard treatment. The treatment prolongs survival and improves clinical, biochemical, and histological features in 65% to 80% of patients [1][2]. Recent studies show that the 10-year survival rate among treated patients may exceed 90%, which is almost identical to that in an age- and sex-matched control population [8]. However, the 20-year survival rate may be less than 80% among patients without cirrhosis and less than 40% among those with cirrhosis at presentation [9]. The reversibility of fibrosis and even cirrhosis in AIH has been recently confirmed in several studies [10][11][12][13]. Given the gap in our current knowledge on long-term patient outcomes and the course of the disease, long-term study of a large number of patients can give us a better understanding of the disease. Herein, we report the long-term outcome in AIH patients after first receiving medical attention for the disease. We also report the nature and rate of clinical, biochemical, and histological responses to treatment and their determinants in a large cohort of AIH patients.

## 2. Objectives

We evaluated the clinical presentation and the natural history of AIH in a large cohort of type I AIH patients from Iran.

## 3. Patients and Methods

All AIH patients who were referred to the outpatient Clinic of the Digestive Disease Research Center (DDRC) of Tehran University of Medical Sciences from 1997 to 2008 were enrolled in this study. Baseline evaluation included a complete history and physical examination, complete blood count (CBC), and a comprehensive biochemical and serological profile, including aspartate aminotransferase (AST), alanine aminotransferase (ALT), alkaline phosphatase (ALP), serum gamma-glutamyl transferase (GGT), serum γ-globulin (GGB), serum albumin, total protein, total and direct bilirubin, erythrocyte sedimentation rate (ESR), prothrombin time, serum creatinine and blood urea nitrogen. Patients were also evaluated for chronic viral hepatitis, Wilson’s disease, α-1 antitrypsin deficiency, and hemochromatosis by using HBsAg, HBcAb, HBsAb, HCV Ab, serum ceruloplasmin, protein electrophoresis, serum iron, and total iron-binding capacity. Diagnosis of AIH was made according to the criteria for the diagnosis of AIH devised in 1993 [5] and its revised version [6]. In brief, AIH was diagnosed if patients had chronically elevated aminotransferases and gamma globulins, with positive antinuclear antibody (ANA), anti-smooth muscle antibody (SMA), or anti-liver–kidney microsomal antibody (LKM-1) type 1, and histopathological features compatible with autoimmune hepatitis on liver biopsy. Patients were only diagnosed with AIH if they did not have viral (hepatitis B and C), metabolic (Wilson’s disease, α-1 antitrypsin deficiency, hemochromatosis), and drug-induced liver disease (including alcohol). Autoantibodies were tested by indirect immunofluorescence. A titer of ≥ 1 : 40 was considered to be positive. HBsAg was determined by Enzyme-Linked Immunosorbent Assay (ELISA; Stat Fax, Awareness Technology Inc., Palm City, FL, USA) using commercially available kits and anti-HCV Ab was assayed using a third generation ELISA test (Ortho-Clinical Diagnostics, Amersham, UK).

Percutaneous liver biopsy was performed for all patients at baseline and for 28 patients who consented to a second biopsy at the end of treatment. Specimens were fixed in 10% formalin; embedded in paraffin; and stained with hematoxylin and eosin, Masson’s trichrome, and reticulin. All specimens were evaluated by a single pathologist. Liver biopsies were considered adequate if there were at least 6 portal tracts per high-power field. Specimens were scored using the modified Hepatitis Activity Index, in which necroinflammation is graded from 0 to 18 and fibrosis from 0 to 6 [14]. Cirrhosis was defined by histology or clinical imaging and laboratory findings of splenomegaly, esophageal varices, ascites, and low platelet counts. Decompensated cirrhosis was defined as the presence of prolonged prothrombin time, emaciation, intractable ascites, and encephalopathy attack. Treatment was started with prednisolone (30 – 50 mg/day). The corticosteroid was then tapered according to patients’ clinical and biochemical response. Treatment continued with the addition of 50 mg azathioprine when the prednisolone dose reached 30 mg. The azathioprine dose was increased to 2 mg/kg body weight when the prednisolone dose decreased to 10 mg. Patients were then maintained on azathioprine, at a dose determined by their biochemical liver profiles and CBCs. If patients were intolerant to azathioprine for any reason, they were maintained on low-dose (5–10 g) prednisolone. Calcium and vitamin D3 supplements were administered to all subjects. Patients who could not tolerate or did not respond to prednisolone for induction therapy were switched to low-dose cyclosporine (3 mg/kg body weight per day, orally) for 6–12 months. Dose adjustments were made to maintain the cyclosporine trough level at 100–300 ng/ml. Cyclosporine was then tapered off and azathioprine maintenance instituted as described above. Ursodeoxycholic acid (UDCA) was added for all patients with overlap AIH/PBC or AIH/PSC and for patients with elevated ALP or GGT that was more than 2 times the normal concentration, despite normalization of transaminases for immunosuppressive therapy. Patients with advanced decompensated inactive cirrhosis were not enrolled in this study and were sent for liver transplant evaluation. Patients with postmenopausal severe osteopenia, emotional instability, poorly controlled hypertension, and brittle diabetes were treated with cyclosporine instead of prednisolone for induction therapy. Azathioprine was avoided in patients with severe pretreatment cytopenia (white blood cell counts below 2500 or platelet counts below 50000) or who developed cytopenia during therapy. Patients underwent clinical and biochemical examinations every 6 weeks during the first 6 months of treatment and every 4 to 6 months after remission. Serum albumin, AST, ALT, ALP, total bilirubin, CBC, and prothrombin time were measured at every visit. An abdominal ultrasound examination was performed at regular intervals for all subjects. During each follow up visit, compliance, response to treatment, and possible drug side effects were recorded. Any complication or major events including esophageal or gastric varices, upper gastrointestinal bleeding, ascites, spontaneous bacterial peritonitis, and encephalopathy were also recorded. Complete remission was defined as disappearance of symptoms and normalization of serum ALT, AST (to equal or less than 30 IU/L), total bilirubin, and immunoglobulin levels with minimal activity in the follow-up liver biopsy (when applicable). Incomplete response was defined as a decrease in the level of liver enzymes by more than half of that observed at the baseline, but that was never less than twice the upper normal limit. Treatment failure was defined as development of clinical complications (i.e., development of ascites, jaundice or hepatic encephalopathy) laboratory deterioration or < 50% decrease in liver enzymes during therapy. Relapse was defined as a rise in serum liver enzymes greater than three-fold the upper normal limit after remission with or without active disease in histology or the reappearance of symptoms.

Continuous variables were expressed as mean and standard deviation (SD) values Dichotomous variables were compared using chi-square tests. P value < 0.05 were considered significant. Paired t tests were used to compare variables before and after treatment. Correlations between quantitative variables and the stage of fibrosis at presentation were assessed using Spearman correlation coefficients. Logistic regression analysis was then used to assess the independent association of various variables with the absence of fibrosis in both the first biopsy, performed at presentation, and in the biopsy performed after treatment. Statistical analysis was performed using SPSS, version 16.0 (SPSS, Inc., Chicago, IL). The study protocol was approved by the Ethics Committee of the Digestive Disease Research Center (DDRC), and informed consent was obtained from all patients prior to enrollment in the study.

## 4. Results

Among the 102 patients enrolled in this study, 35 and 67 fulfilled IAIHG pre-therapy criteria for definite and probable AIH, respectively. One patient had both AIH and hepatitis C. Twenty-seven patients (26.4%) had overlap syndrome, of whom 19 (18.6%) had AIH/PBC and 8 (7.8%) had AIH/PSC. Seventy-five patients (73.5%) were women. Women comprised 76.0%, 84.2%, and 25.0% of AIH, AIH/PBC, and AIH/PSC patients, respectively (AIH vs. AIH/PSC P = 0.004, and AIH/PBC vs. AIH/PSC P < 0.001). The mean age was 29.1 ± 15.3 years at the time of disease onset and 29.6 ± 15.1 years at the time of diagnosis (range, 3–63 years). Thirty-three patients (32.3%) were older than 40 years and 5 (4.9%) were younger than 10 years. The age distribution showed 2 peaks: one among 11–20 year-old patients and the other among 41–45 year-old patients. There was no difference in the mean age of patients with the 3 diagnostic categories of AIH. Forty-two patients (41.3%) had histologic cirrhosis (stage 5 or 6) at presentation. Acute presentation was observed in 43 patients (42.2%) and was defined as the presence of recent onset (< 30 days) symptoms (jaundice and/or drowsiness), in conjunction with ALT levels higher than 10 times the upper normal limit and elevated prothrombin time (> 15 s). AIH patients and patients with overlap syndrome did not differ in their response to treatment or progression to cirrhosis. The most common symptoms in descending order were as follows: jaundice (73%), fatigue (66%), abdominal pain (23%), pruritis (20%), arthralgia (16%), fever (14%), and weight loss (3%). At the time of physical examination, splenomegaly and ascites were detected in 58.4% and 16.9% of the patients, respectively. Sixteen women (21.3%) reported menstrual abnormalities. One patient was seropositive for anti-HCV antibodies with very low titers of HCV RNA (HCV-RNA = 250 copy / l). Ninety patients (88.2%) were seropositive for ANA and/or SMA and 1 patient showed anti-LKM-1 antibodies (type II AIH). ANA was the most frequent autoantibody detected (50.0%). Thirty-eight patients (39.5%) had other autoimmune conditions in addition to AIH. The most frequent condition was thyroiditis (16%), followed by diabetes (10%), ulcerative colitis (9%), and rheumatoid arthritis (3%). Baseline demographic, biochemical, and histological data are presented in (Table 1). In the pre-treatment liver biopsies, we found no fibrosis (stage 0) in 1 patient (0.9%), portal fibrotic expansion (stage 1-2) in 13 patients (12.7%), bridging fibrosis (stage 3-4) in 46 patients (45.1%), and cirrhosis (stage 5-6) in 42 patients (41.3%). Cirrhotic patients were, on an average, approximately 6 years younger than non-cirrhotic patients (P = 0.047). Patients younger than 20 years of age were more likely to be cirrhotic at presentation than those older than 45 years of age (70.5% vs. 46.6%, P = 0.016). Serum γ–globulin and PT were significantly higher in cirrhotic patients than in non-cirrhotic patients (P = 0.002 and P = 0.019, respectively). No other demographic, baseline clinical, or biochemical data were associated with histological fibrosis or cirrhosis at presentation. We detected cirrhosis in the baseline biopsies of 37 patients who presented with splenomegaly (61.6%).

**Table 1:** Baseline Demographic, Biochemical, and Histological Data for 102 Patients With Autoimmune Hepatitis. All values are Mean ± SD, otherwise noted.

	**Overall**	**Cirrhotic**	**Non-Cirrhotic**	**P value b**
Female, No. (%)	75 (73.5)	42 (72.4)	33 (75.0)	0.77^c^
Age at presentation, y	29.1 ± 15.3	26.5 ± 15.2	32.5 ± 14.8	0.049
AST a, IU/L	524.9 ± 428.9	540.0 ± 414.4	513.9 ± 442.6	0.77
ALT a, IU/L	552.8 ± 480.1	605.9 ± 481.5	514.7 ± 479.7	0.36
Alkaline phosphatase, IU/L	441.7 ± 285.4	444.8 ± 300.1	439.3 ± 276.6	0.93
Serum γ –globulin, g/dL	255.4 ± 219.6	367.6 ± 252.3	172.3 ± 148.1	0.002
Total bilirubin, mg/dL	5.8 ± 6.8	7.4 ± 8.4	4.6 ± 4.9	0.06
Direct bilirubin, mg/dL	3.7 ± 4.6	4.9 ± 5.7	2.7 ± 3.2	0.02
Total protein, g/dL	8.2 ± 1.3	8.0 ± 1.4	8.4 ± 1.1	0.32
Albumin, g/dL	2.5 ± 1.5	2.3 ± 1.5	2.9 ± 1.6	0.08
White blood cell, No./mm^3^	7628 ± 3682	8682 ± 4458	6848 ± 2781	0.06
Hemoglobin, g/dL	12.6 ± 1.8	12.6 ± 1.9	12.8 ± 1.6	0.65
ESR a	37.2 ± 31.0	33.1 ± 31.6	42.3 ± 30.1	0.22
Platelet count (× 1000), No./mm^3^	199.5 ± 111.0	183.9 ± 121.4	220.7 ± 92.5	0.14
Prothrombin time, s	15.6 ± 3.7	16.1 ± 2.9	14.9 ± 4.6	0.16
INR a	1.7 ± 0.9	1.8 ± 0.8	1.5 ± 1.2	0.28
Blood urea nitrogen, mg/dL	14.1 ± 7.3	15.0 ± 8.4	13.3 ± 6.4	0.45
Creatinine, mg/dL	0.8 ± 0.4	0.9 ± 0.6	0.7 ± 0.3	0.09
HAI a	12.7 ± 3.9	14.5 ± 3.1	10.9 ± 3.9	< 0.001
Grade	9.1 ± 3.1	9.8 ± 2.8	8.4 ± 3.4	0.049
Stage	3.9 ± 1.3	4.8 ± 0.9	2.9 ± 1.0	< 0.001

^a^ Abbreviations: ALT, alanine aminotransferase; AST, aspartate aminotransferase; ESR, erythrocyte sedimentation rate; HAI, hepatitis activity index; INR, international normalized ratio

^b^ P value calculated using t test

^c^ P value calculate using chi square test

**Figure 1:**
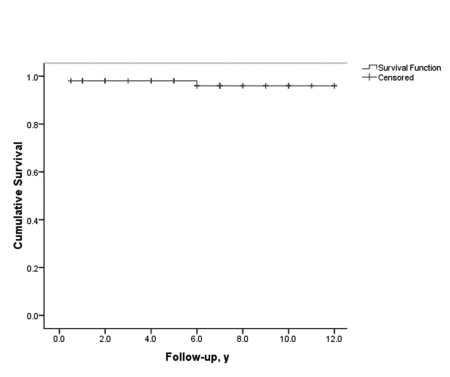
Ten-Year Survival Curve for 102 Autoimmune Hepatitis Patients

**Figure 2:**
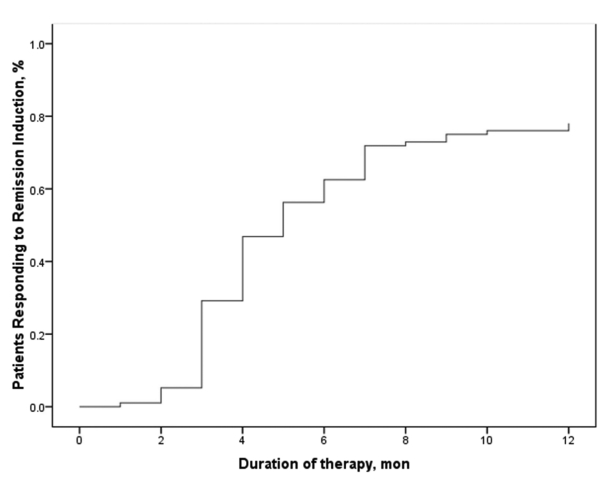
Time Course of Remission Induction in Autoimmune Hepatitis Patients Undergoing Treatment. The majority of patients experienced complete remission within three months Of starting therapy

**Figure 3:**
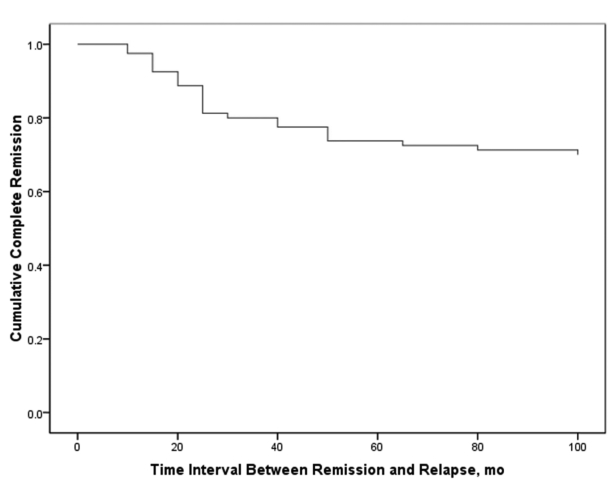
Time Course of Relapse Among Patients Who Responded to Remission Induction

Mean duration of follow-up was 60.0 ± 38.4 months (range, 12–144 months). Follow-up was regular in 71 patients (69.6%) and irregular in 31 (30.4%). Overall, 80 patients achieved remission (79.4%) and 22 (21.6%) either responded partially or did not respond to treatment. Among the 80 patients who achieved remission, 27 (33.7%) developed stable and well-compensated cirrhosis while in biochemical remission. Of the 22 subjects with treatment failure, 6 underwent orthotopic liver transplantation and 3 died of liver failure while on the transplant list (Figure 1). The remaining 13 patients who did not respond to treatment were in the decompensated phase of cirrhosis and on supportive therapy while they awaited liver transplantation. Sixteen (72.7%) of the 22 patients who did not respond to therapy had irregular follow up and were noncompliant with their medications. The main reason for noncompliance was a change in facial appearance following corticosteroid therapy, especially among younger individuals. Only 5 patients required low-dose prednisolone in addition to azathioprine for maintenance therapy. In 22 patients (29.3%), induction therapy was switched to cyclosporine because of poor adherence and response or corticosteroid intolerance. Among these patients, treatment was successful in 9, ended in stable cirrhosis in 10, and failed in 3. Side effects were mainly related to corticosteroids and consisted of moon face (33.3%), acne (27.3%), striae (18.2%), gingivitis (18.2%), hypertrichosis (9.1%), and hyperglycemia (3.0%). One patient developed generalized sepsis and died. Majority of the patients (87%) complained of bone pain after corticosteroid therapy. The main side effect of azathioprine was a reduction in white blood cell and platelet counts. This occurred in 5.8% of the patients and was managed by decreasing the dose or switching to corticosteroid maintenance. All patients tolerated the low dose of cyclosporine very well, with occasional side effects such as hirsutism. There were no occurrences of major side effects such as renal failure. The ultimate outcome of immunosuppressive therapy in AIH patients is summarized in Tabulation. The 10-year survival rate in all 102 AIH patients was 96%. The mean interval between the initiation of treatment and remission was 3.6 ± 2.1 months. After 3 months of therapy, 50 patients (63.3%) achieved remission, while 24 patients (30.4%) achieved remission after 3–6 months of treatment and 5 (6.3%) achieved remission after 6–12 months of treatment. One patient responded after 12 months of treatment (Figure 2). The mean time interval between initiation of therapy and remission was higher in the overlap groups than in the AIH group (5.5 ± 3.7 vs. 3.5 ± 1.8 months, respectively; P = 0.048). Of the 80 patients who achieved remission, 24 (30.0%) had at least one relapse while on maintenance therapy (Figure 3). If normal ALT and AST levels for remission were defined as lower than 30 IU/L in men and lower than 19 IU/L in women (instead of the normal upper limit provided by the laboratory), then 21 of those who relapsed (87.5%) had abnormal liver enzymes at the time of remission and 35 of the 56 patients who did not relapse (63.6%) had abnormal liver enzymes at remission (P = 0.003 and P = 0.004 for ALT and AST, respectively). Mean interval between remission and the first relapse was 27.1 ± 15.6 months. Gender did not affect remission rate, but patients who achieved remission were, on an average, older than those who did not respond to treatment (31.3 ± 15.5 vs. 21.0 ± 11.3 years, respectively; P = 0.005). None of the demographic, clinical or biochemical variables (other than AST and ALT) at the time of remission predicted relapse. A second liver biopsy was performed in 28 patients who consented to the procedure (18 patients treated with prednisolone, 8 treated with cyclosporine, and 2 overlap syndrome patients receiving UDCA and prednisolone). The mean interval between the 2 biopsies was 4.7 ± 2.8 years. In the follow-up biopsies, we found the following: no fibrosis (stage 0) in 4 patients (14.3%), portal fibrotic expansion (stage 1-2) in 12 patients (42.9%), bridging fibrosis (stage 3-4) in 9 patients (32.1%), and cirrhosis (stage 5-6) in 3 patients (10.7%). The mean stage score of the second biopsy decreased by 1.8 ± 1.0 from the mean score of the first biopsy (P = 0.002). Fibrosis scores decreased by 1–3 points in 18 patients (64.2%), did not change in 8 patients (28.6%), and worsened by 1 point in 2 patients (7.1%). Among the 18 patients in whom fibrosis decreased, 12 had a 1-point improvement, 4 had a 2-point improvement, and 2 had a 3-point improvement. In 8 of the 10 patients who were cirrhotic at presentation and underwent post-treatment biopsy, fibrosis decreased by 1–3 points. Fibrosis did not get worse in any of the non-cirrhotic patients. In both overlap syndrome patients who underwent a second biopsy, the fibrosis score was worse for the second biopsy than for the first biopsy. The first of these patients had AIH/PBC and the fibrosis score increased from 1 to 2. The second patient had AIH/PSC, and the fibrosis score increased from 2 to 4 over the course of treatment. Treatment failed in both cases. Mean inflammation score decreased by 2.6 ± 3.7 points after treatment. Inflammation improved in 17 patients (60.7%), did not change in 7 patients (25%), and worsened in 4 patients (14.3%). The mean decrease in the grade of inflammation among those who improved was 5.1 ± 2.5 points. All 18 patients (100%) who experienced regression of fibrosis, showed at least 2-point improvement in their hepatic inflammatory score, while only 1 of the 10 patients (10%) with no improvement in fibrosis showed an improved inflammatory score (1-point improvement; P < 0.001). The mean inflammatory grade decreased by 4.6 ± 3.1 points for patients with improved fibrosis after treatment, while the mean inflammatory grade increased by 0.6 ± 3.1 points for patients with no fibrosis improvement (P < 0.001). Both inflammation and fibrosis correlated with ALT levels but not with AST levels (Table 2). According to univariate analysis, the 8 AIH patients treated with cyclosporine showed more fibrosis improvement between their first and second biopsy than the 18 patients treated with prednisolone (P = 0.023). In addition, patients who experienced improved fibrosis had a mean interval of 68.4 months between the first and last biopsies, while those who did not experience improved fibrosis had a mean interval of 32.4 months between biopsies (P = 0.005). When examined using multivariate logistic regression, however, the only variable associated with improved fibrosis regression was a longer interval between the 2 biopsies (OR = 2.15, 95% CI: 1.11-4.16, P = 0.023). None of the demographic, baseline clinical, or biochemical data before or after treatment was significantly associated with histological fibrosis or cirrhosis after treatment.

**Tabulation:** Final Outcome for 102 Autoimmune Hepatitis Patients at the End of the Follow-Up Period (Mean 5 Years)

**Final Outcome**	**Patients, No. (%)**
Complete remission	53 (52.0)
Compensated cirrhosis	27 (26.5)
Treatment failure (decompensated cirrhosis)	13 (12.7)
Liver transplantation	6 (5.9)
Death	3 (2.9)
Total	102 (100)

**Table 2:** Correlations Between Biochemical and Histological Improvement in 102 Patients With Autoimmune Hepatitis

	**CorrelationCoefficient**	**P value b**
** Inflammation Improvement**		
Decrease in ALT a	0.50	0.03
Decrease in AST a	0.43	0.06
** Fibrosis Improvement**		
Decrease in ALT	0.46	0.04
Decrease in AST	0.23	0.33

^a^ Abbreviations: ALT, alanine aminotransferase; AST, aspartate aminotransferase

^b^ P value is calculated by Pearson correlation

## 5. Discussion

Several characteristics of our patients were quite similar to those of AIH patients in other case series reports. These characteristics include the bimodal age distribution of patients at presentation; clinical, biochemical, and histopathological findings at presentation; presence of concurrent autoimmune diseases; and response to corticosteroids [15]. Twenty-seven patients (26.4%) in our study had overlap syndrome, and AIH/PBC was more common among the patients in our study than in those of other reported series. In our study, 17% of the patients had AIH/PBC and 8% had AIH/PSC , while in Japan only 2% of AIH patients had PBC [16] and in an Indian study only 1 patient (1.1%) had overlap syndrome [17]. In our study, 42 patients (41.3%) were cirrhotic at presentation; this finding is similar to that of previous reports [8][18]. Serum γ–globulin and PT were significantly higher in cirrhotic patients than in non-cirrhotic patients. Moreover, the cirrhotic patients were generally younger than the non-cirrhotic patients. As in previous studies, none of the baseline demographic or biochemical parameters examined by us predicted fibrosis or cirrhosis at presentation [16][18]. Overall, 78.4% of the patients achieved initial remission. As in previous studies, the majority of the patients who responded to treatment (94%) did so within 6 months [8]. During maintenance therapy, 30% of the patients had at least one relapse. Cirrhosis at presentation did not affect treatment outcome. Majority of the patients tolerated prednisolone and azathioprine very well, and those who were intolerant or non-responsive responded to and tolerated low dose of cyclosporine very well. Therefore, low dose of cyclosporine can be used as an alternative to corticosteroids for induction of remission in problematic AIH patients, especially in young women and patients with osteoporosis. Using multivariate logistic regression, only platelet count and PT were recognized as independent predictors of non-response to treatment. This finding is similar to that observed in our previous study [11]. Therefore, AIH patients with a persistently low platelet count and prolonged PT in spite of therapy need to be counseled appropriately about the likely outcomes of their disease and the possible need for future liver transplantation. Our data also show that fibrosis improves at a slow pace and follow-up biopsies at short time intervals may be misleading in this regard. Our data confirms that AIH responds promisingly to appropriate immunosuppressive therapy. The patients in our study showed a 10-year survival rate of 96%; this rate is comparable to that observed in age- and sex- matched control populations [8]. This high survival rate was achieved despite the fact that 41% of the patients were cirrhotic at presentation. The main reason for treatment failure was poor adherence to induction therapy because of changes in the facial appearance of young subjects who are still in school or college or are planning to marry who found their facial appearance to be embarrassing and unacceptable. Alternatives to high-dose corticosteroids may help improve compliance. Considering its safety profile, our data, and the data of others, low-dose cylosporine may be a good candidate [19]. We recently completed a randomized controlled head-to-head comparison of low-dose cyclosporine with prednisolone for AIH induction therapy and found that low-dose cyclosporine is as effective as prednisolone, but less toxic [20]. In addition to cyclosporine, other calcineurin inhibitors like tacrolimus [21][22] and purine antagonists like mycophenolate mofetil [21] have been used successfully to treat corticosteroid-resistant AIH patients. In a randomized controlled trial, budesonide has recently been shown to be as effective as prednisolone, with fewer side effects [23]. Although induction of remission was successful in a substantial percentage of patients, relapse was quite common, ranging from 30% after 6 months to 70% after 2–3 years of follow-up [24][25]. An important finding in our study was that decreasing AST/ALT levels to less than 30 IU/L for men and less than 19 IU/L for women (instead of reducing AST/ALT levels to less than twice the normal value reported by the laboratory) lessened the risk of relapse. Other investigators have also reported this phenomenon [18][26]. Long-term continuation of maintenance therapy for those in remission can also prevent relapse [18][26]. In conclusion, wise use of current treatment methods for AIH will help most patients. The main reason for rapid disease progression, liver failure, and cirrhosis in AIH patients is unsuitable/deferred therapy or noncompliance with the prescribed therapeutic regimen. Multiple strategies may be considered to improve outcomes for AIH patients, including early diagnosis, targeting AST and ALT values below 30 IU/L for men and 19 IU/L for women, early identification of problematic patients, and close follow-up to find and promptly treat patients who experience relapse.

## References

[1] Czaja AJ (1984). Natural history, clinical features, and treatment of autoimmune hepatitis.. Semin Liver Dis.

[2] Krawitt EL (2006). Autoimmune hepatitis.. N Engl J Med.

[3] Longhi MS, Ma Y, Mitry RR, Bogdanos DP, Heneghan M, Cheeseman P, Mieli-Vergani G, Vergani D (2005). Effect of CD4+ CD25+ regulatory T-cells on CD8 T-cell function in patients with autoimmune hepatitis. J Autoimmun.

[4] Czaja AJ (1996). The variant forms of autoimmune hepatitis. Ann Intern Med.

[5] Johnson PJ, McFarlane IG (1993). Meeting report: International Autoimmune Hepatitis Group. Hepatology.

[6] Alvarez F, Berg PA, Bianchi FB, Bianchi L, Burroughs AK, Cancado EL, Chapman RW, Cooksley WG, Czaja AJ, Desmet VJ, Donaldson PT, Eddleston AL, Fainboim L, Heathcote J, Homberg JC, Hoofnagle JH, Kakumu S, Krawitt EL, Mackay IR, MacSween RN, Maddrey WC, Manns MP, McFarlane IG, Meyer zum Büschenfelde KH, Zeniya M (1999). International Autoimmune Hepatitis Group Report: review of criteria for diagnosis of autoimmune hepatitis. J Hepatol.

[7] Carpenter HA, Czaja AJ (2002). The role of histologic evaluation in the diagnosis and management of autoimmune hepatitis and its variants. Clin Liver Dis.

[8] Kanzler S, Lohr H, Gerken G, Galle PR, Lohse AW (2001). Long-term management and prognosis of autoimmune hepatitis (AIH): a single center experience. Z Gastroenterol.

[9] Roberts SK, Therneau TM, Czaja AJ (1996). Prognosis of histological cirrhosis in type 1 autoimmune hepatitis. Gastroenterology.

[10] Malekzadeh R, Mohamadnejad M, Nasseri-Moghaddam S, Rakhshani N, Tavangar SM, Sohrabpour AA, Tahaghoghi S (2004). Reversibility of cirrhosis in autoimmune hepatitis. Am J Med.

[11] Mohamadnejad M, Malekzadeh R, Nasseri-Moghaddam S, Hagh-Azali S, Rakhshani N, Tavangar SM, Sedaghat M, Alimohamadi SM (2005). Impact of immunosuppressive treatment on liver fibrosis in autoimmune hepatitis. Dig Dis Sci.

[12] Cotler SJ, Jakate S, Jensen DM (2001). Resolution of cirrhosis in autoimmune hepatitis with corticosteroid therapy. J Clin Gastroenterol.

[13] Dufour JF, DeLellis R, Kaplan MM (1997). Reversibility of hepatic fibrosis in autoimmune hepatitis. Ann Intern Med.

[14] Ishak K, Baptista A, Bianchi L, Callea F, De Groote J, Gudat F, Denk H, Desmet V, Korb G, MacSween RN (1995). Histological grading and staging of chronic hepatitis. J Hepatol.

[15] Czaja AJ, Manns MP (2010). Advances in the diagnosis, pathogenesis, and management of autoimmune hepatitis. Gastroenterology.

[16] Omagari K, Kinoshita H, Kato Y, Nakata K, Kanematsu T, Kusumoto Y, Mori I, Furukawa R, Tanioka H, Tajima H, Koga M, Yano M, Kohno S (1999). Clinical features of 89 patients with autoimmune hepatitis in Nagasaki Prefecture, Japan. J Gastroenterol.

[17] Amarapurkar DN, Patel ND (2007). Spectrum of autoimmune liver diseases in western India. J Gastroenterol Hepatol.

[18] Czaja AJ (2007). Autoimmune hepatitis. Part B: diagnosis. Expert Rev Gastroenterol Hepatol.

[19] Malekzadeh R, Nasseri-Moghaddam S, Kaviani MJ, Taheri H, Kamalian N, Sotoudeh M (2001). Cyclosporin A is a promising alternative to corticosteroids in autoimmune hepatitis. Dig Dis Sci.

[20] Nasseri-Moghaddam S, Karimian S, Khashayar P (2009). Cyclosporine A versus Prednisolone for Induction of Remission in Autoimmune Hepatitis: A Randomized Controlled Trial. Hepatology.

[21] Aqel BA, Machicao V, Rosser B, Satyanarayana R, Harnois DM, Dickson RC (2004). Efficacy of tacrolimus in the treatment of steroid refractory autoimmune hepatitis. J Clin Gastroenterol.

[22] Chatur N, Ramji A, Bain VG, Ma MM, Marotta PJ, Ghent CN, Lilly LB, Heathcote EJ, Deschenes M, Lee SS, Steinbrecher UP, Yoshida EM (2005). Transplant immunosuppressive agents in non-transplant chronic autoimmune hepatitis: the Canadian association for the study of liver (CASL) experience with mycophenolate mofetil and tacrolimus. Liver Int.

[23] Manns MP, Woynarowski M, Kreisel W, Lurie Y, Rust C, Zuckerman E, Bahr MJ, Günther R, Hultcrantz RW, Spengler U, Lohse AW, Szalay F, Färkkilä M, Pröls M, Strassburg CP; European AIH-BUC-Study Grou (2010). Budesonide induces remission more effectively than prednisone in a controlled trial of patients with autoimmune hepatitis. Gastroenterology.

[24] Hegarty JE, Nouri Aria KT, Portmann B, Eddleston AL, Williams R (1983). Relapse following treatment withdrawal in patients with autoimmune chronic active hepatitis. Hepatology.

[25] Montano-Loza AJ, Carpenter HA, Czaja AJ (2007). Improving the end point of corticosteroid therapy in type 1 autoimmune hepatitis to reduce the frequency of relapse. Am J Gastroenterol.

[26] Montano-Loza AJ, Carpenter HA, Czaja AJ (2007). Consequences of treatment withdrawal in type 1 autoimmune hepatitis. Liver Int.

